# Low-Temperature Adaptation of the Snow Alga *Chlamydomonas nivalis* Is Associated With the Photosynthetic System Regulatory Process

**DOI:** 10.3389/fmicb.2020.01233

**Published:** 2020-06-10

**Authors:** Yanli Zheng, Chunling Xue, Hui Chen, Chenliu He, Qiang Wang

**Affiliations:** ^1^Key Laboratory of Algal Biology, Institute of Hydrobiology, Chinese Academy of Sciences, Wuhan, China; ^2^University of Chinese Academy of Sciences, Beijing, China; ^3^State Key Laboratory of Crop Stress Adaptation and Improvement, School of Life Sciences, Henan University, Kaifeng, China; ^4^Innovation Academy for Seed Design, Chinese Academy of Sciences, Wuhan, China

**Keywords:** antioxidant enzymes, *Chlamydomonas nivalis*, cyclic electron transfer, low temperature, photosynthesis

## Abstract

The alga *Chlamydomonas nivalis* thrives in polar snow fields and on high-altitude mountain tops, and contributes significantly on primary production in the polar regions, however, the mechanisms underlying this adaptation to low temperatures are unknown. Here, we compared the growth, photosynthetic activity, membrane lipid peroxidation, and antioxidant activity of *C. nivalis* with those of the model alga *C. reinhardtii*, under grow temperature and low temperatures. *C. nivalis* maintained its photosynthetic activity in these conditions by reducing the light-harvesting ability of photosystem II and enhancing the cyclic electron transfer around photosystem I, both of which limited damage to the photosystem from excess light energy and resulted in ATP production, supporting cellular growth and other physiological processes. Furthermore, the increased cyclic electron transfer rate, carotenoid content, and antioxidant enzyme activities jointly regulated the reactive oxygen species levels in *C. nivalis*, enabling recovery from excess excitation energy and reduced photooxidative damage to the cell. Therefore, we propose a model in which adaptive mechanisms related to photosynthetic regulation promote the survival and even blooming of *C. nivalis* under polar environment, suggesting that *C. nivalis* can provide organic carbon sources as an important primary producer for other surrounding life in the polar regions for maintaining ecosystem.

## Introduction

The eukaryotic and prokaryotic single-celled organisms known as microalgae are important inhabitants of ocean, freshwater, and even terrestrial ecosystems (Msanne et al., 2012; Li et al., 2016). The efficient photosynthesis of microalgae provided the basic conditions required for all complex life on Earth (Zhang et al., 2014; Chen et al., 2015b; Li et al., 2016). Microalgae are ideal model organisms due to their fast growth, strong adaptability to extreme environments, and high oil contents.

Snow algae grow in the polar snow fields and in high-altitude mountain tops. Most of the algae in polar snow algal community belong to Chlorophyceae, Chlamydomonadales or Volvocales, some of which have received more attention in recent years, such as *Chlamydomonas nivalis* (Cvetkovska et al., 2016), *Chlamydomonas* cf. *nivalis* strain CCALA 970 (Lukes et al., 2014), *Chloromonas reticulate* (Matsuzaki et al., 2012), *Sanguina nivaloides*, and *Sanguina aurantia* (Procházková et al., 2019). *C. nivalis*, belonging to the genera *Chlamydomonas* (Chlorophyta) and closely relating to the model algae *Chlamydomonas reinhardtii*, is a typical snow alga and a leading system for investigating cold adaptation (Cvetkovska et al., 2016). *C. nivalis* produces substantial biomass even under extreme conditions, such as low temperature, high light, low pH, nutrient deficiency, freeze-thaw cycles, and UV irradiation, and thus serves as a vital food source for other cold-adapted organisms, such as ice worms, collembola, and bacteria (Thomas and Duval, 1995; Ursula et al., 1996; Painter et al., 2001).

*C. nivalis* cells possess specialized mechanisms that allow them to withstand extreme environmental stresses, such as a high accumulation of lipids and carotenoids, a reduced number of light-harvesting pigment–protein complexes, and high levels of astaxanthin esterified with fatty acids, which reduces light damage and photoinhibition, maintaining maximum photosynthesis efficiency (Yong and Lee, 1991; Bidigare et al., 1993; Rezanka et al., 2014; Hulatt et al., 2017). Nonetheless, the adaptive mechanisms by which *C. nivalis* withstands low temperatures are unclear.

Photosynthesis, which converts carbon dioxide into chemical energy using energy from sunlight, is the major mechanism by which most photosynthetic organisms harvest energy (Liang et al., 2013). Photosynthesis takes place in the thylakoid membrane and involves a four-subunit protein complex comprising photosystem II (PSII), photosystem I (PSI), the cytochrome b_6_/f (Cyt b_6_f) complex, and ATP synthase (Hohmann-Marriott and Blankenship, 2011). PSII, PSI, and Cyt b_6_f are connected in a linear electron transfer (LET) chain and couple proton pumping with ATP synthesis via ATP synthase (Zhan et al., 2016). Around PSI, two types of electron transfer exist: LET, which generates ATP and NADPH, and cyclic electron transfer (CET), which generates ATP at times of NADPH shortage (Yamori et al., 2015). CET regulates the balance of ATP/NADPH in photosynthetic cells and protects the light system from high levels of light damage. Under low temperatures, NDH-dependent CET plays an important role in relieving oxidative damage in chloroplasts in photosynthetic organisms (Shikanai, 2007; Yamori et al., 2011; Zhang et al., 2013).

When photosynthetic organisms are exposed to stress, the rate of photosynthesis decreases and excess electrons are transferred to molecular oxygen (O_2_) to form reactive oxygen species (ROS) (Mittler, 2002; Liu et al., 2017). ROS include ^1^O_2_, H_2_O_2_, O^2–^, and HO**.**, which cause oxidative damage to proteins, DNA, and lipids (Apel and Hirt, 2004; Song et al., 2014; Chen et al., 2015a). The scavenging system of ROS includes antioxidant enzymes [such as superoxide dismutase (SOD), catalase (CAT), and peroxidase (POD)] and non-enzymatic scavengers [such as carotenoids, Vitamin E (V_E_), and Vitamin C (V_C_)] (Edreva, 2005; Szivak et al., 2009; Zhao Q. et al., 2018).

To elucidate the adaptive mechanisms by which *C. nivalis* survives low temperatures, we investigated the cell growth, photosynthetic activity, and antioxidant mechanisms of this alga. In contrast to the model green alga *C. reinhardtii, C. nivalis* grows well in low temperatures by maintaining a normal level of photosynthetic activity. Moreover, the CET rate in *C. nivalis* rapidly rose in cold temperatures, which reduced the damage caused by excess light, while the activities of the antioxidant enzymes were also dramatically enhanced, mitigating the effects of excess ROS production. All above adaptive mechanisms promote the survival and even blooming of *C. nivalis* under polar environment.

## Materials and Methods

### Algal Cultures

*Chlamydomonas reinhardtii* and *Chlamydomonas nivalis* strains were purchased from Chlamydomonas Resource Center^1^ and UTEX Culture Collection of Algae^2^, respectively. *C. nivalis* (UTEX 2824) and *C. reinhardtii* were grown in TAP medium, at temperatures of 4, 12, and 22°C with a light intensity of 100 μmol m^–2^ s^–1^. The cell biomass was recorded using a cell counter (Z1 Dual Beckman Coulter, United States), and the cell size was observed using a fluorescence microscope (Olympus BX53, Japan).

### Pigment Quantifications

Measurement of chlorophyll content followed Chen et al. (2018) and Guan et al. (2018) with some modifications. After 72 h culture of algal cells (10^6^ cells ml^–1^) grown at 22°C and treated by turning from 22 to 4°C (similarly hereinafter), algal cells were precipitated, respectively, by centrifugation at 4,000 rpm for 5 min at 22 and 4°C. The supernatant was discarded and the pellet was resuspended in 80% acetone, overnight at 4°C, and then centrifuged at 12,000 rpm for 3 min at room temperature. A spectrophotometer was used to determine the concentrations of various photosynthetic pigments using the following formulae (Lichtenthaler, 1987):

Chlorophyll a (Chl a) (mg ml^–1^) = 12.25 A_663.2_ − 2.79 A_646.8_;
Chlorophyll b (Chl b) (mg ml^–1^) = 21.50 A_646.8_ − 5.10 A_663.2_;
Total chlorophylls (Chl a+b) (mg ml^–1^) = 7.15 A_663.2_ + 18.71 A_646.8_;
Total carotenoids (mg ml^–1^) = (1,000 A_470_–1.82 Chl a − 85.02 Chl b)/198.

### Chlorophyll Fluorescence Analysis

Chlorophyll fluorescence was quantified using a Dual-PAM-100 Chl fluorescence photosynthesis analyzer (Walz, Germany). Intact cells from 22 and 4°C culture were adapted for 15 min in the dark at 22 and 4°C, respectively, prior to measuring their initial (Fo) and maximum (Fm) fluorescence levels. The maximum quantum yield of PSII photochemistry was calculated as Fv/Fm = (Fm − Fo)/Fm, as described by Geller et al. (2018). The parameter 1-qL, which estimates the proportion of closed PSII reaction centers or the excitation pressure of PSII, was calculated using the following formula: 1-qL = 1 − [(Fm′ − F′) / (Fm′ − Fo′) × (Fo′/F′)]. The effective photochemical quantum yield of PSII was calculated as Y (II) = (Fm′ − F)/Fm (Kramer et al., 2004). The non-regulated energy dissipation Y (NO) was calculated as Y(NO) = F/Fm. The regulated energy dissipation Y (NPQ) was calculated as Y (NPQ) = F/Fm′ − F/Fm (Kramer et al., 2004). Y (ND), the non-photochemical energy dissipation caused by donor-side limitations, was calculated as Y (ND) = (P − Po)/Pm. Transient increases in chlorophyll fluorescence after turning off actinic light (AL) were detected as described by Shikanai et al. (1998).

### Electron Transfer Rates

The electron transfer rates of PS II, PS I, and CET were monitored using a Clark-type oxygen electrode (Oxylab 2, Hansatech, United Kingdom) at 22 and 4°C, as described by Wang et al. (2008) and Chen et al. (2015a). White light was provided at an intensity of 500 μmol m^–2^ s^–1^. Thylakoid membranes were used for all measurements, and the chlorophyll contents were adjusted to 15 mg ml^–1^. The PSI reaction mixture included 40 mM methylviologen (MV), 5 mM NH_4_Cl, 2 mM ascorbic acid, 0.1 mM 2,6-dichlorophenolindophenol (DCPIP), 2 mM NaN_3_, 40 μM 3-(3,4-dichlorophenyl)-1,1-dimethylurea (DCMU), 40 mM tricine (pH 7.5), and 100 mM sucrose. The electrons in the mixture were transferred from DCPIP/ascorbic acid via PSI to MV. One oxygen molecule was consumed for each electron transport event. The PSII reaction mixture contained 5 mM NH_4_Cl, 4 mM K_3_FeCN, 1 mM phenyl-*p*-benzoquinone (BQ), 40 mM tricine (pH 7.5), and 100 mM sucrose and was used to measure the electron transport from H_2_O via PSII to BQ. One oxygen molecule was produced for every four electrons transported. The CET reaction mixture contained 10 μM 3-(3,4-dichlorophenyl)-1,1-dimethylurea (DCMU), 0.5 mM NADPH, 10 mM NaCl, 5 mM MgCl_2_, 10 mM KCl, 0.25 mM KH_2_PO_4_, and 2 mM NaCl ethylene diamine tetraacetic acid (EDTA), 1 mM MnCl_2_, and 50 mM 4-(2-hydroxyethyl)-1-piperazineethanesulfonic acid (HEPES, pH 7.6) and was used to measure the electron transport from NADPH via PSI to O_2_. One oxygen molecule was consumed for each electron transport event. DCMU inhibited the transfer of electrons from PSII. The evolution/consumption of O_2_ was followed for 3 min, and the rate, V, was calculated as follows: electron transport rates (μmol O_2_ mg^–1^ Chl a h^–1^) = V × 60 × 1,000/30.

### Immunoblot Assays

Immunoblot detection was performed as described (Hu et al., 2014; Zhang H. et al., 2018; Zhao X. et al., 2018) with some modifications. Proteins for the SDS-PAGE immunoblot analysis were extracted using 40 mM Tris-HCl (pH 8.0) and then quantified using a BCA Kit (Tiangen, China). Equivalent concentrations of protein for each sample were denatured in 5 × SDS-PAGE loading buffer. Rabbit primary antibodies and goat anti-rabbit secondary antibodies (Sigma-Aldrich, United States) were diluted with skim milk powder (1:3,000 and 1:6,000, respectively). The hybridized proteins were detected with chemiluminescence. Antibody against CAS was produced in rabbits as described by Chen et al. (2015a). Antibodies against D1 and CP43 were produced in rabbits, and the antibody preparation and verification of antibodies specificity were showed in Supplementary Figure S1. The quantification of CAS, D1, and CP43 protein was performed using ImageJ (ver1.41, NIH) (Tsihlis et al., 2010).

### Real-Time PCR Analysis

Real-time PCR analysis was performed as described (Zhao et al., 2013, 2015; Chen et al., 2017a; Dong et al., 2018; Zhang J. et al., 2018) with some modifications. Cells were harvested from solutions containing 10^7^ cells ml^–1^ by centrifugation at 3,000 rpm for 5 min. The precipitate was resuspended in a 1.5 ml Eppendorf tube containing 1 ml TRIZOL reagent (Thermo Fisher Scientific, United States), precipitated in 100% isopropanol, and washed in 75% ethanol. The resulting RNA pellet was suspended in 20 μl DEPC water, after which the RNA was quantified using a NanoDrop (Thermo Fisher Scientific). Aliquots were stored at –70°C.

The expression of *CAT*, *SOD*, *POD*, and a gene encoding the chloroplast-localized Ca^2+^ sensor (CAS) was analyzed using real-time PCR (Ursula et al., 1996). First-strand cDNA was generated using a PrimeScript RT Reagent Kit with a gDNA Eraser, following the manufacturer’s instructions (#RR047A, Takara Bio, Japan). Specific primers were designed to produce 100- to 200-bp PCR products (Supplementary Table S1) using 18S rRNA and CBLP as references. Quantitative real-time PCR was performed using the 2 × iTaq Universal SYBR Green Supermix (#172, Bio-Rad Laboratories, United States) by a Bio-Rad CFX96 Thermal Cycler. Differences in expression were calculated by melt-curve analysis using Bio-Rad CFX manager software v3.0 (Bio-Rad). The PCR conditions were as follows, 95°C for 1 min followed by 40 cycles of 95°C for 5 s, 55°C for 30 s. The melting curve was completed by 65–95°C and 5 s increased by 0.5°C.

### Assessment of Lipid Peroxidation and ROS Scavenging Enzyme Activities

Malondialdehyde (MDA) levels and CAT, POD, and SOD activities were measured as described by Zhang et al. (2013) with some modifications. Cells (10^7^ cells ml^–1^) were harvested by centrifugation at 4,000 *g* for 5 min, after which the cell pellet was washed with ultrapure water and resuspended in 0.2 M sodium phosphate buffer (pH 7.8). The resuspended cells were broken using an ultrasonic cell disruptor (Scientz, China) and then centrifuged at 1,800 *g* for 10 min at 4°C. The supernatants were used to analyze MDA levels and enzyme activities. Their protein contents were assayed using a BCA Protein Quantification Kit (Tiangen Biotech, China). The MDA levels and CAT, POD, and SOD activities were measured using an MDA Assay Kit (Beyotime, China), CAT Activity Assay Kit (Beyotime), POD Activity Assay Kit (Nanjing Bioengineering Institute, China), and SOD Activity Assay Kit (Beyotime), respectively, according to the manufacturer’s instructions. The MDA level (nM mg^–1^ protein) was calculated as nanomoles of MDA per milligram of protein. CAT activity (U mg^–1^ protein) was defined as the amount of enzyme that caused a micromole reduction in H_2_O_2_ each second per milligram of cellular protein at 37°C. POD activity (U mg^–1^ protein) was defined as the amount of enzyme that catalyzed a milligram of substrate each minute per milligram of cellular protein at 37°C. SOD activity (U mg^–1^ protein) was defined as the amount of enzyme that caused a 50% decrease of the SOD-inhabitable NBT per milligram of cellular protein at 37°C.

### Thermoluminescence Measurements

High-temperature thermoluminescence (HTL) was measured by Thermoluminescence System TL 400/PMT (Photon Systems Instruments, Brno, Czech Republic). Fifty microliter suspensions of *C. reinhardtii* and *C. nivalis* cells (10^9^ cells) were acquired by filtration (0.45 μm, GE Healthcare Life Sciences, United States) pressed against copper film, dark-adapted for 10 min at 20°C, and cooled at 10°C for 1 min. The HTL emissions of the samples were recorded while the suspensions were warmed from 10 to 160°C (heating rate: 0.1°C s^–1^). N_2_ gas was used to desiccate samples and prevent any oxidation induced by high temperatures. The instruments were driven by a computer, using a specially developed acquisition program. Detailed instructions can be obtained elsewhere (Ducruet, 2003; Guerrero et al., 2014).

### 77 K Fluorescence Spectroscopy

Fluorescence emission spectra at 77 K were measured using an F-4700 Fluorescence Spectrophotometer (Hitachi) fitted with a liquid nitrogen cold-finger dewar. Both the excitation and emission widths were 5 nm. The excitation wavelength was 430 nm. Samples were adjusted to a Chl concentration of 15 μg ml^–1^. Data were normalized at 687 nm.

### Statistical Analysis

Four or five biological replicates were performed for each experiment. All data were analyzed using SPSS ver19, with the significance level set to a confidence interval of 95 or 99%. A *t*-test was applied to evaluate the means and SD of replicated studies. The differences between the control and test values were measured using a one-way ANOVA test, and statistical differences were determined as *P* < 0.05 or *P* < 0.01.

## Results

### *C. nivalis* Adapts to Low Temperatures

**FIGURE 1:**
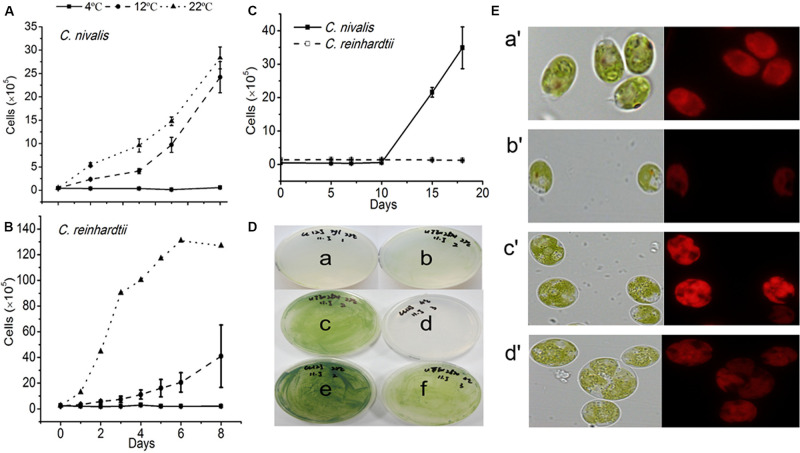
The growth rates and states of *C. nivalis* and *C. reinhardtii* at different temperatures. **(A,B)** Growth curves of *C. nivalis* and *C. reinhardtii* under static culture at 4, 12, and 22°C. **(C)** Growth curves of *C. nivalis* and *C. reinhardtii* under shaking culture at 4°C. **(D)** *C. nivalis* and *C. reinhardtii* cells cultured on a solid medium at 22 and 4°C. (a,b) Growth of *C. reinhardtii* and *C. nivalis*, respectively, after 0 days. (c,f) Growth of *C. nivalis* for 20 days at 22 and 4°C, respectively. (d,e) Growth of *C. reinhardtii* for 20 days at 4 and 22°C, respectively. **(E)** Changes in cell size and chlorophyll fluorescence based on assessment of 10^5^ cells under 22°C and 4°C. (a′,b′), Changes in *C. reinhardtii* after 20 days at 22 and 4°C, respectively. (c′,d′) Changes in *C. nivalis* after 20 days at 22 and 4°C, respectively. All data points in the current and following figures represent the means and SD of four or five biological replicates (*t-*test, *p*< 0.05).

The growth of *C. reinhardtii* and *C. nivalis* cells was evaluated at three temperatures (4, 12, and 22°C) to determine the temperature tolerance of the two strains. *C. nivalis* showed a slightly reduced growth at 12°C compared to its growth at 22°C (Figure 1A), but *C. reinhardtii* severely retarded growth at 12°C (Figure 1B). Both strains failed to grow at 4°C when cultured statically (Figures 1A,B). However, *C. nivalis* cells with stationary culture always stuck to the reactor wall, which is not conducive to cell growth. Therefore, shaking culture was used in subsequent culture. *C. nivalis* showed a rapid increase in cell growth after the 10th day of shaking culture at 4°C. Although *C. reinhardtii* cultured at 4°C didn’t die for a long time, it consistently failed to grow (Figure 1C). This indicates that *C. nivalis* adapted to the low temperatures, but *C. reinhardtii* was more seriously affected by the cold. Therefore, in the following experiments, 22 and 4°C were chosen as the control and low-temperature conditions.

*C. nivalis* and *C. reinhardtii* growth on solid media was consistent with the results described above (Figure 1D). In addition, while *C. reinhardtii* displayed a diminished cell size based on assessment of 10^5^ cells and much weaker chlorophyll autofluorescence at 4°C than at 22°C, *C. nivalis* showed a slightly reduced chlorophyll fluorescence but increased cell size at low temperatures (Figure 1E). The increase in cell size helps *C. nivalis* to defense against cold with increased cell content, and decreased surface/volume ratio (which means lowered contact with cold). These observations suggest that the larger cell size of *C. nivalis* at low temperatures may increase its ability to withstand such conditions.

### *C. nivalis* Maintains Robust Photosynthetic Activity at Low Temperatures

Photosynthesis plays an important role in the cellular growth of microalgae and is often affected by adverse conditions. To evaluate the effects of low temperature on photosynthesis, we determined the intracellular chlorophyll contents of *C. nivalis* and *C. reinhardtii* at 4°C. In *C. nivalis*, the contents of both Chl a and Chl b were not significantly affected by the low temperatures, while the photoprotective pigments, carotenoid content, and the Chl a/Chl b ratio, the representative of the reaction center to antenna ratio increased significantly, which may contribute to the low temperature tolerance of this species (Table 1). By contrast, although the carotenoid content and Chl a/Chl b ratios of *C. reinhardtii* were dramatically increased at low temperatures, the contents of both Chl a and Chl b were significantly reduced, indicating serious damaging effects of low temperatures on photosynthetic pigments.

**TABLE 1:** The pigment contents of *C. nivalis* and *C. reinhardtii* measured at 22 and 4°C.

	*Chlamydomonas nivalis*		*Chlamydomonas reinhardtii*	
	4°C	22°C	4°C	22°C
Chl a (10^–6^ μg cell^–1^)	1.54 ± 0.32	1.49 ± 0.42	0.599 ± 0.17	1.1 ± 0.05
Chl b (10^–6^ μg cell^–1^)	0.49 ± 0.11	0.79 ± 0.21	0.31 ± 0.07	1.11 ± 0.05*
Chl a/Chl b	3.14 ± 0.21	1.88 ± 0.08*	1.95 ± 0.12	1.88 ± 0.22
Car (10^–5^ μg cell^–1^)	5.4 ± 0.55	0.13 ± 0.03**	0.91 ± 0.37	0.03 ± 0.003

Chl a, Chlorophyll a; Chl b, Chlorophyll b; Car, Carotene. All data represent the means and SD of four or five biological replicates (t-test, p < 0.05). The significance of the differences between 4 and 22°C in the same strain were tested using a one-way ANOVA. *p < 0.05, **p < 0.01.

**FIGURE 2:**
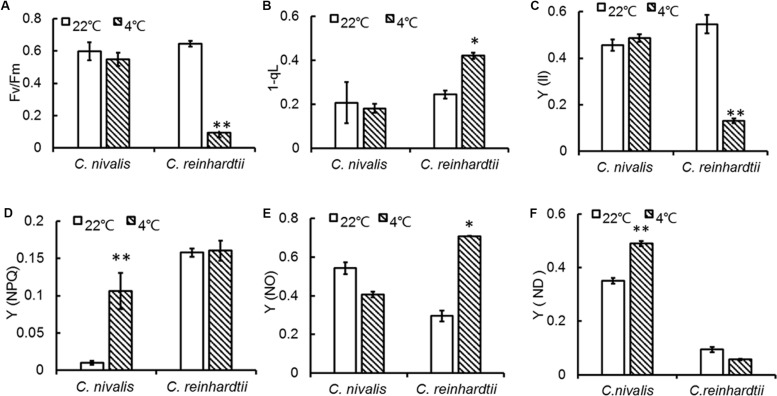
PSII changes in *C. nivalis* and *C. reinhardtii* at low temperatures. **(A)** Fv/Fm, the maximum quantum yield of PSII photochemistry. **(B)** 1-qL, the proportion of closed PSII centers. **(C)** Y (II), the effective photochemical quantum yield of PSII. **(D)** Y (NPQ), regulated energy dissipation. **(E)** Y (NO), non-regulated energy dissipation. **(F)** Y (ND), non-photochemical energy dissipation due to donor-side limitation. The significance of the differences between 22 and 4°C in the same strain in the current and following figures were tested using a one-way ANOVA. *p < 0.05, **p < 0.01.

Fv/Fm represents the maximum quantum yield of PSII photochemistry, and a decrease in Fv/Fm can directly reflect the effect of stresses on photosynthesis (Demetriou et al., 2007; Zhang et al., 2013). A change in chlorophyll fluorescence was determined for *C. reinhardtii* under low temperature, however, the Fv/Fm of *C. nivalis* was not significantly affected (Figure 2A). The 1-qL value indicates the proportion of closed PSII centers, which is related to PSII activation. Y (II) represents actual quantum yield of PSII in any illumination levels. The 1-qL and Y (II) values of *C. nivalis* remained stable under low temperatures, unlike those of *C. reinhardtii* (Figures 2B,C). All these results indicate that *C. nivalis* could maintain good photosynthetic activity at low temperatures. By contrast, the rapid decreases of Fv/Fm and Y (II) and the significant increase of 1-qL revealed the inactivation of PSII in *C. reinhardtii* during the severe stress inflicted at low temperatures.

During photosynthesis, some of the unused light energy is dissipated. Y (NPQ) reflects the dissipation of excess light energy in the form of heat, representing photoprotection (Nama et al., 2018). Y (NO) indicates the excess light energy not lost to heat dissipation, representing photodamage (Christof Klughammer, 2008; Erhard and Schreiber, 2008; Nama et al., 2018). The Y (NPQ) of *C. nivalis* was markedly increased at low temperatures compared to higher temperatures (Figure 2D), while Y (NO) was dramatically increased in *C. reinhardtii* but slightly decreased in *C. nivalis* (Figure 2E). These results indicate that PSII of *C. nivalis* was not damaged at low temperatures and that the excess light energy was dissipated as heat rather than photodamage, thereby maintaining PSII activity.

**FIGURE 3:**
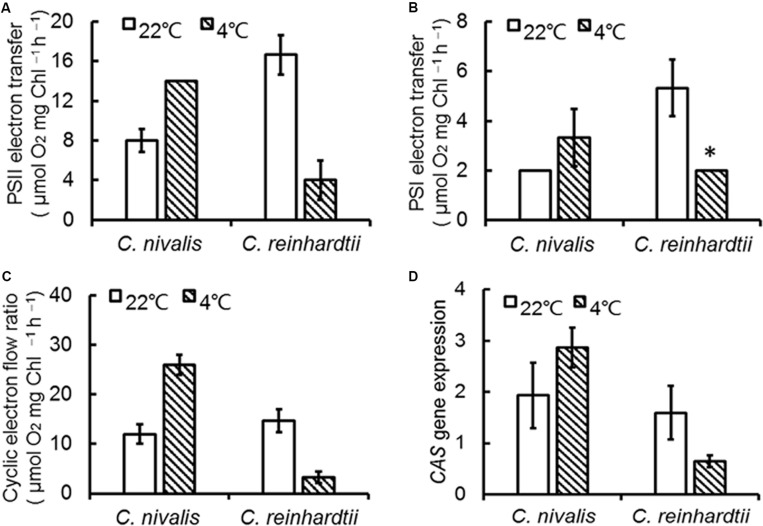
Electron transfer rate of *C*. *nivalis* and *C. reinhardtii* at 22 and 4°C. **(A)** PSII electron transfer. **(B)** PSI electron transfer. **(C)** Cyclic electron transfer. **(D)** Expression of *CAS* using real-time fluorescence quantitative PCR. The significance of the differences between 22 and 4°C in the same strain were tested using a one-way ANOVA. *p < 0.05, **p < 0.01.

Y (ND) represents the quantum yield of donor side-limited heat dissipation in PSI, which is induced by the downregulation of Cyt b_6_f activity and/or PSII damage. The value of Y (ND) in *C. nivalis* dramatically increased at low temperatures, while for *C. reinhardtii*, it decreased (Figure 2F). The above results revealed that PSI activation was undamaged in *C. nivalis*. The electron transfer rates around PSI and PSII were further detected to assess photosynthetic activity (Figure 3). While the PSII electron transfer, PSI electron transfer, and CET were blocked in *C. reinhardtii* under low temperatures, the electron transfer rate around PSI and PSII, and particularly the CET, of *C. nivalis* increased at low temperatures, further highlighting the stable photosynthetic system function in this species. Also, the expression of the gene encoding CAS, a protein involved in CET, was upregulated in *C. nivalis* at 4°C (Figure 3D).

**FIGURE 4:**
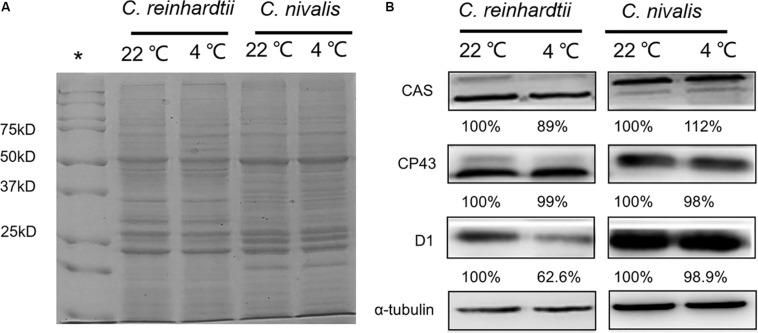
Protein level and relative protein abundance. **(A)** Representative image of SDS-PAGE analysis of total proteins extracted from *C. nivalis* and *C. reinhardtii* at 22 and 4°C as the loading control in the immunoblot analysis. A 20 μg aliquot of proteins from each sample was separated on a 12% SDS-PAGE gel and stained with Coomassie Brilliant Blue. **(B)** Relative protein abundances of the CAS, CP43, D1, and reference protein α-tubulin. *: Standards.

Furthermore, we determined the protein levels of the PSII central proteins (CP43 and D1) to evaluate the effects of low temperature on PSII. The levels of CP43 and D1 in *C. nivalis* were similar at 4 and 22°C, and the levels of D1 were significantly higher in *C. nivalis* than *C. reinhardtii* at both temperatures (Figure 4B), indicating that low temperature does not affect the integral PSII proteins of *C. nivalis*, and CP43 in *C. nivalis* may have been post-translationally modified in *C. nivalis*, potentially for the adaptation of this species to low temperatures. The possible post-translational modification (PTM) was also detected in the CAS protein in *C. nivalis*, while the increased level of CAS in this species indicated its improved CET at low temperatures, both of which may have facilitated its adaptation to low temperatures (Figure 4B).

### CET Around PSI and ROS-Scavenging Mechanisms Play Important Roles in the Adaptation to Low Temperatures

**FIGURE 5:**
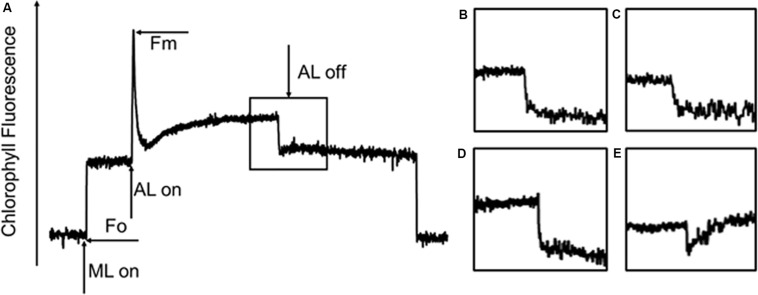
Chlorophyll fluorescence analysis of *C. nivalis* and *C. reinhardtii* at 22 and 4°C. **(A)** Transient post-illumination increase in Chl fluorescence. ML, measure light; AL, actinic light; Fo, initial fluorescence level; Fm, maximum fluorescence level. The curve exhibits a representative trace of Chl fluorescence in *C. reinhardtii* at 22°C. The black box represents cells exposed to AL (100 μE) for 5 min, after which the AL was turned off. Short-lived increases in chlorophyll fluorescence were detected under low ML. **(B–E)**. Magnified traces from the boxed area. **(B)** *C. reinhardtii* at 22°C. **(C)** *C. reinhardtii* at 4°C. **(D)** *C. nivalis* at 22°C. **(E)** *C. nivalis* at 4°C.

**FIGURE 6:**
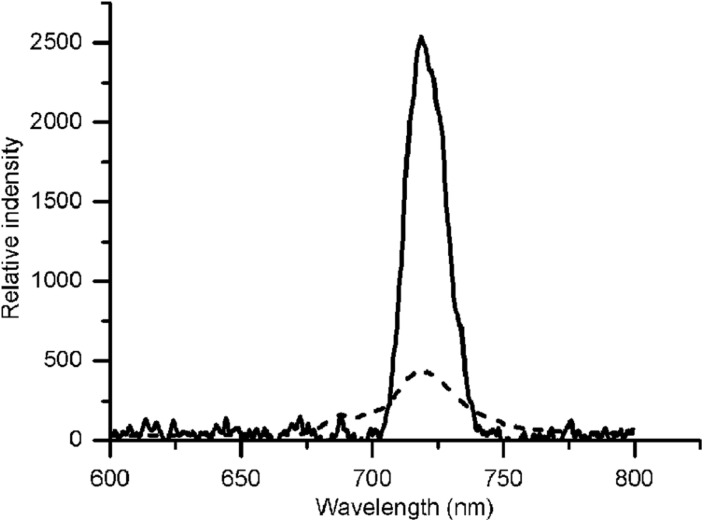
77 K fluorescence emission spectra of *C. nivalis* at 22°C and 4°C. Broken line, 22°C- *C. nivalis.* Solid line, 4°C- *C. nivalis.*

Our previous studies revealed that the rate of CET around PSI increased significantly, thereby supplementing ATP, in an adaptive response to environmental stress in *Chlorella* (Zhang et al., 2013; Chen et al., 2015a). Some studies pointed out the transient rise in chlorophyll fluorescence was due to the reduction of PQ under dark conditions, which was dependent on the activity of NDH, so fluorescence dark rise is indicating the activity of NDH (Kotera et al., 2005). NDH is a multi-subunit complex embedded in the thylakoid membrane that participated in the CET around the PSI (Shikanai, 2007) suggesting the indication of CET by NDH activity. In addition, NDH is also involved in chlororespiration (Desplats et al., 2009). Therefore, the transient fluorescence increase in the dark has been reported as a tool to investigate the CET around PSI or the level of chlororespiration (Houille-Vernes et al., 2011; Peng et al., 2011). *C. reinhardtii* displayed a typical chlorophyll fluorescence curve at 22°C (Figure 5A). *C. nivalis* showed an increase in chlorophyll fluorescence at 4°C after termination of AL (Figures 5D,E), however, no increase was detected in *C. reinhardtii* (Figures 5B,C), suggesting the increase of NDH activity in *C. nivalis*, which can be influenced by CET or chlororespiration. Furthermore, for determining the change of CET more intuitively, the electron transfer rate of CET was measured using oxygen electrode by providing electron donors and receptors, and the results clearly showed that the CET of *C. nivalis* increased at low temperatures (Figure 3C). 77 K fluorescence emission spectra of *C. nivalis* at 22°C showed a major peak at 722 nm (*F722*), which corresponded to PSI, and a smaller peak at 687 nm (*F687*), which originated mainly from PSII (Figure 6). However, the increased *F722* but decreased *F687* at 4°C indicated that PSII was more susceptible at low temperature, but PSI content were significantly enhanced for responding to low temperature. The increased *F722* at low temperature correspondence with the increased CET around PSI measured (Figures 3C, 5E). All these results indicate that the CET ratio of *C. nivalis* was significantly enhanced at low temperatures, suggesting a mechanism that resists stress by enhancing CET at low temperatures.

Higher Chl a/Chl b ratios could decrease the collection of light in relation to the rate of PSII photochemistry, while the increased carotenoid content of *C. nivalis* had an antioxidative role in trapping excess light that would otherwise be absorbed by the chloroplasts (Table 1). Both of these factors could impact ROS accumulation; therefore, we next measured the lipid peroxidation levels and antioxidant enzyme activities to monitor the level of ROS in the cells. The lipid peroxidation level was evaluated by examining the MDA content and high-temperature thermoluminescence (HTL) of the algae.

**FIGURE 7:**
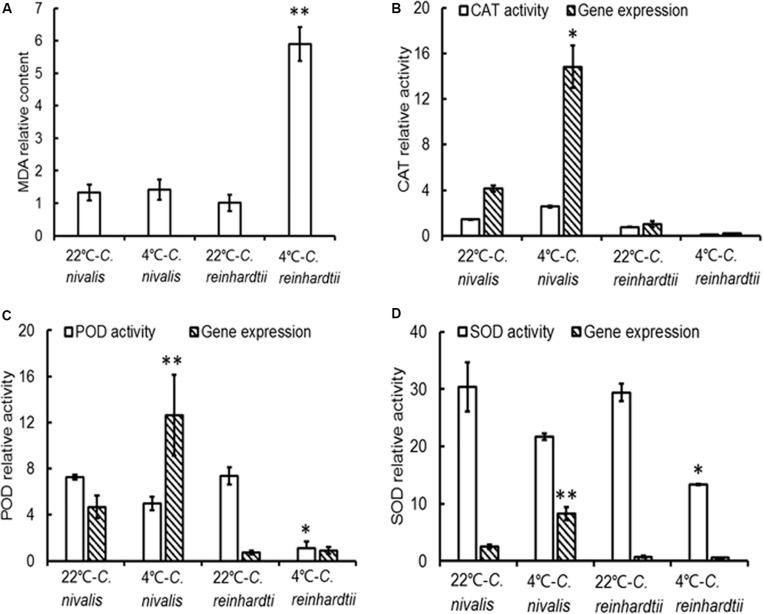
MDA levels, antioxidant enzyme activities, and gene expression in *C. nivalis* and *C. reinhardtii* at 22 and 4°C. **(A)** MDA content (mg prot ml^–1^). **(B)** CAT activity (U mg^–1^ protein s^–1^) and expression levels. **(C)** POD activity (U mg^–1^ prot) and expression levels. **(D)** SOD activities (U mg^–1^ prot) and expression levels. The significance of the differences between 22 and 4°C in the same strain were tested using a one-way ANOVA. *p < 0.05, **p < 0.01.

**FIGURE 8:**
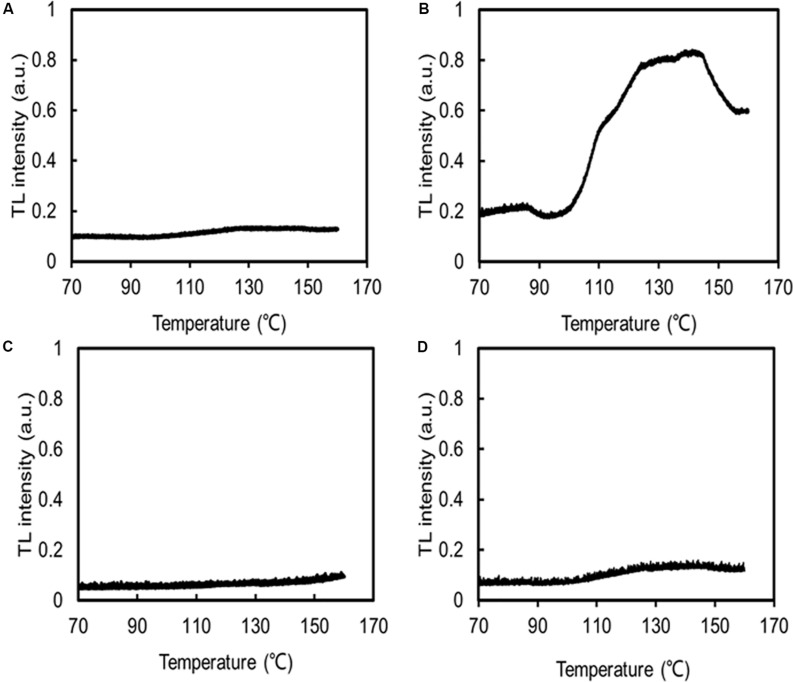
HTL glow curves indicating lipid peroxidation. The same concentrations of *C. nivalis* and *C. reinhardtii* were measured. Samples were heated from 10 to 160°C, at a rate of 0.1°C s^–1^, and their emissions were subsequently recorded. N_2_ gas was flushed through the samples to dry them and prevent any oxidation induced by high temperatures, a.u. arbitrary units. **(A)** *C. reinhardtii* at 22°C. **(B)** *C. reinhardtii* at 4°C. **(C)** *C. nivalis* at 22°C. **(D)** *C. nivalis* at 4°C.

The MDA contents of *C. reinhardtii* were significantly increased under low temperatures, but the MDA content of *C. nivalis* at 4°C was similar to that at 22°C (Figure 7A), indicating no accumulation of ROS. HTL measurements also reflect the level of lipid peroxidation in photosynthetic organisms (Havaux, 2003). Increasing the temperature from 120 to 140°C can be used to assess the specificity of the band, which represents the level of lipid peroxidation due to triplet carbonyls and singlet oxygens (Vavilin and Ducruet, 1998; Ducruet, 2003; Havaux, 2003). A broad HTL peak at around 135°C represents the level of lipid peroxidation, with taller peaks indicating more extensive lipid peroxidation. At 4°C, *C. reinhardtii* exhibited very high levels of lipid peroxidation, but the lipid peroxidation level in *C. nivalis* was not significantly elevated (Figures 8B,D), suggesting that *C. nivalis* possessed effective ROS scavenging mechanisms under low temperatures. This may also be an important element for the adaptation of *C. nivalis* to low temperatures.

**FIGURE 9:**
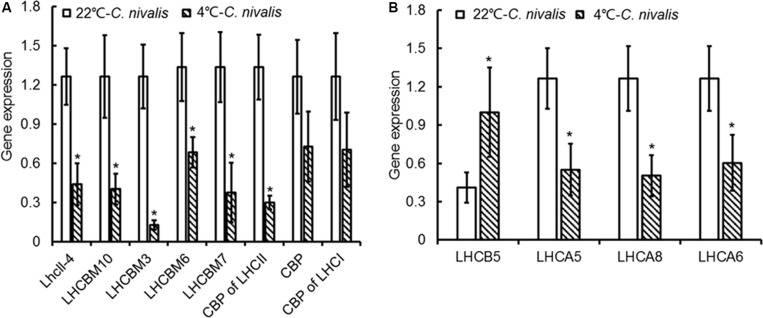
Expression of genes related to light-harvesting in *C. nivalis* at 22°C and 4°C. **(A)** Chlorophyll-binding protein in light-harvesting complex. **(B)** Chlorophyll-binding protein in photosystem. LhcII-4, light-harvesting chlorophyll-a/b binding protein; LHCBM10, light-harvesting complex II protein m10; LHCBM3, light-harvesting complex II chlorophyll-a/b binding protein M3; LHCBM6, chlorophyll-a/b binding protein of LHCII type I, chloroplast precursor; CPB of LHCII, chlorophyll-a/b binding protein of LHCII; CPB, chlorophyll-a/b binding protein; CPB of LHCI, chlorophyll-a/b binding protein of LHCI; LHCB5, light-harvesting chlorophyll-a/b binding protein of PSII; LHCA5, light-harvesting chlorophyll-a/b binding protein of PSI; LHCA8, light-harvesting chlorophyll-a/b binding protein of PSI; LHCA6, light-harvesting chlorophyll-a/b binding protein of PSI. The significance of the differences between 22 and 4°C in the *C. nivalis* were tested using a one-way ANOVA. *p < 0.05, **p < 0.01.

The expression level of genes related to light-harvesting in *C. nivalis* at 22 and 4°C were detected to evaluate the light-harvesting ability of photosystem (Figure 9). It is found that gene expression levels of chlorophyll-binding protein in light-harvesting complex and PSI were decreased significantly at low temperature. *C. nivalis* cells were more susceptible to light stress at low temperature, and the reducing light energy absorption can effectively reduce light damage and enhance light protection to avoid ROS accumulation. Furthermore, we evaluated the antioxidant enzyme activities and gene expression levels at low temperatures. Compared with *C. reinhardtii*, the activity and corresponding gene expression levels of CAT, POD, and SOD were dramatically increased in *C. nivalis* at 4°C (Figure 7). In particular, the CAT activity increased markedly in *C. nivalis* from 22 to 4°C (Figure 7B). These antioxidant enzymes may have effectively cleared the intracellular ROS produced in *C. nivalis* under low temperatures and maintained cellular activity.

In addition, the increased gene expression of *POD* and *SOD* in *C. nivalis* under low temperatures contrasted with their reduced enzymatic activity (Figures 7C,D), suggesting that POD and SOD were regulated at the transcriptional level.

## Discussion

All environmental conditions influence the metabolic pathways in cells. Specialized microalgae culture methods are more susceptible to external conditions, which affect the metabolism of the cultured cells. *C. nivalis* is an unusual *Chlamydomonas* in that it can survive and grow well when exposed to cold temperatures (Figure 1), indicating that this species possesses special mechanisms that allow it to adapt to cold environments. This is consistent with the results of Geller et al. (2018), who showed that snow algae possess higher growth rates than typical algae under highlight and low temperatures.

As an important driver of cell growth and other physiological processes in photosynthetic organisms, photosynthesis is often adversely affected under stress conditions. For example, we previously showed that nitrogen starvation, high levels of nitrite, and high levels of ammonia could substantially inhibit the photosynthesis and respiration of *Chlorella* (Zhang et al., 2013; Zhao et al., 2014; Chen et al., 2015a, 2016; Mo et al., 2015; Li et al., 2016; Wang et al., 2018). Maintaining good levels of photosynthetic activity under stress conditions is a key factor enabling microalgae to adapt to stress conditions. In addition, Johnson and Alric (2012) showed that the acetate in TAP medium had the impact on the dark redox state of the chloroplast in *C. reinhardti*. Both inorganic and organic carbon are contained in TAP medium, indicating that two *Chlamydomonas* strains in this study grew mixotrophically. Heifetz et al. (2000) also pointed out that mixotrophy with acetate did not affect algal growth, PSII efficiency, chlorophyll content and respiration of *Chlamydomonas*. In this study, we focused on the detection and compare of photosynthetic system activity including algal growth, PSII efficiency and chlorophyll content (Figures 1–4 and Table 1), so the addition of acetate would not interference on the results.

Degrenne et al. (2010) reported that when *Chlamydomonas* cells were culture mixotrophically with organic carbon and inorganic carbon together, acetate was initially utilized as an organic carbon source and was almost completely exhausted after about 4 day, and then inorganic carbon was utilized subsequently. In this study, two *Chlamydomonas* strains were cultured at normal temperature for 5 day, and the acetate might completely consumed before low temperature culture of 72 h, which should be few additional effect on the changes of photosynthetic activity. In addition, Terauchi et al. (2010) pointed out that organic carbon in the medium could ensure steady respiration when photosynthesis was affected. Form Figure 1C, it was observed that when *C. nivalis* with low cell density was cultured directly at low temperature, photosynthesis and cell activity were largely suppressed and respiration might be maintained by acetate for subsequent recovery. Therefore, it is suggested that *C. nivalis* needs the presence of organic matter to help it adapt to low temperature, and snow algae may form a mutually beneficial symbiotic relationship with other microorganisms in their original habitat, by promoting the recycling of limited nutrients via various metabolic ways.

In this study, we showed that, unlike the severe damage caused to the photosynthetic system in *C. reinhardtii* by low temperatures, *C. nivalis* maintained a robust level of photosynthetic activity under low temperatures, as shown by its sustained levels of cell growth, photosynthetic pigment concentrations, chlorophyll fluorescence, electron transfer rate, and photosystem-related proteins and gene expression (Figures 1–4 and Table 1). In addition, this species displayed an increased ratio of Chl a/Chl b, caused by the changes in the ratio between the central protein of PSII and the peripheral light-harvesting proteins, and the decrease of genes expression related to light-harvesting; that is, the light-harvesting ability of PSII and PSI decreased, indicating that *C. nivalis* reduced light damage by reducing light-harvesting to adapt to low temperatures (Table 1 and Figure 9). Y (II) and Y (NPQ) were significantly increased in the *C. nivalis* cells at low temperatures, while 1-qL and Y (NO) were decreased, indicating that *C. nivalis* could increase its light-protective ability by increasing the dissipation of excess light energy as heat and by reducing the light-harvesting capacity of PSII to maintain typical levels of photosynthesis and protect the cell (Figure 2).

Protein PTMs are a vital cellular control mechanism for modulating the diverse properties of proteins and regulating their function (Hart and Ball, 2013). Among the hundreds of different PTMs, lysine acetylation is a highly dynamic and tightly regulated PTM. CP43 is the main subunit involved in the light-harvesting activity of PSII, as well as in the electron transfer reactions. In a previous study, we found that CP43 was acetylated in the cyanobacterium *Synechocystis* sp. PCC 6803 and that this lysine acetylation played a regulatory role in photosynthesis under high-light conditions (Mo et al., 2015). In addition, phosphorylation is an important protein PTM. One study showed that the level and function of CAS under high light were both regulated by phosphorylation and that the higher the light level, the more significant the phosphorylation of CAS (Vainonen et al., 2008). This phosphorylated protein therefore indicates the light changes in the chloroplasts and transmits signals through direct protein–protein interactions, playing a role in the signal cascade reaction coordinating plant growth and the response to environmental stress (Vainonen et al., 2008). In this study, possible protein PTMs might be also observed in CP43 and CAS in *C. nivalis* (Figure 4), suggesting that phosphorylation or lysine acetylation took effect in the transmission of stress signals and the corresponding regulation of the photosynthetic process, contributing to the adaptation of this species to low temperatures. D1 is the primary target of photooxidative damage and is rapidly degraded and replaced by the *de novo* biosynthesis of subunits.

In a study by Warner et al. (1999), PSII activity was significantly decreased, with a significant decline in the D1 protein content due to its greater rate of degradation than *de novo* biosynthesis. They also pointed out that increasing the conversion rate of the D1 protein could significantly reduce photoinhibition. High D1 protein contents could effectively delay PSII degradation under stress, maintaining photosynthesis and prolonging carbohydrate biosynthesis (Scoma et al., 2012). In this study, the decrease in D1 protein levels observed in *C. reinhardtii* were consistent with the significant decrease in PSII activity observed in these cells (Figures 2, 4). By comparison, the expression levels of the D1 protein in *C. nivalis* were relatively stable both at 4 and 22°C; therefore, *C. nivalis* effectively maintained robust photosynthetic activity under low temperatures (Figures 2, 4).

CET plays an important role in enabling photosynthetic organisms to tolerate or adapt to adverse conditions (Zhao et al., 2014), with the basic function of balancing the ratio between NADPH and ATP. Even under normal conditions, this CET function is indispensable for C3 plants (Yamori and Shikanai, 2016). Some studies have shown that, under nitrogen deficiency, CET was gradually enhanced to supplement ATP and regulate the ATP/NADPH balance during the reduction of photophosphorylation and respiratory phosphorylation in *Chlorella* (Zhang et al., 2013; Chen et al., 2014, 2015a; Li et al., 2016). In this study, the transient rise of chlorophyll fluorescence in the dark, which can be influenced by CET or chlororespiration, was used to evaluate the response process of *C. nivalis* at low temperature (Shikanai, 2007; Desplats et al., 2009). Segura and Quiles (2015) demonstrated that chlororespiration and other routes of electron input to the electron transfer chain was probably essential when plants are subjected to intense freeze damage, suggesting the important role of chlororespiration in coping with environmental stress. The fluorescence dark rise in *C. nivalis* suggests that CET, chlororespiration or both play roles in responding to low temperatures (Figures 5D,E). Oxygen electrode was also applied in measurement of the electron transfer rate of CET by providing electron donors and receptors, which clearly showed the increase in CET rate of *C. nivalis* at low temperatures (Figure 3C). Thus, the increased CET rate in *C. nivalis* under low temperatures may contribute to ATP supplement and the consumption of light energy. The excess excitation energy in this species could therefore be recovered and the ATP/NADPH balance could be regulated to protect PSI and avoid ROS accumulation and membrane lipid peroxidation. In addition, the possible role of chlororespiration in the response of *C. nivalis* to low temperature should be further confirmed.

Besides the increased CET rate, the increased carotenoid content and the activity of the antioxidant enzymes in *C. nivalis* under low temperatures may also have played a role in ROS scavenging, as demonstrated by the reduced ROS levels revealed by the results of the MDA and HTL analyses (Figures 7, 8 and Table 1). In oxygenic photosynthetic organisms, most stress conditions can ultimately be described as oxidative stresses, which generate adverse effects on cell physiological function (Elstner, 1991). The ROS scavenging mechanisms above contributed to the maintenance of robust photosynthesis levels in *C. nivalis* and its adaptation to low temperatures by protecting these cells from ROS damage induced by the adverse conditions.

Microalgae can be virtually found everywhere close the surface of the planet (Chen et al., 2019). From the hot equator to the perennially frozen polar regions, algae can be found in seas, rivers, lakes, wet surfaces, rocks, even deserts, and snow. Photosynthetic microalgae are the main contributors to primary production, and algal photosynthesis accounts for almost more than half of the total global primary productivity (Iriarte and Purdie, 1994; Chen et al., 2017b). Especially in a particular ecological environment, microalgae are important primary producers that provide nutrients to other native organisms through photosynthesis (Pulz et al., 2000; Mock and Thomas, 2005; Gattuso et al., 2006). In polar regions, severe environment and climatic conditions limit the spread of terrestrial higher plants, and cold-loving or cold-tolerant algae become the most important photosynthetic primary producers in polar habitats, and form the basis of polar carbon cycle. Williams et al. (2003) detected the photosynthesis of the snow alga community and found that the alga community could fix 2300 μmol CO_2_ m^–2^ day^–1^, and about 3.1 g CO_2_ per month. With the adaptive mechanisms related to photosynthetic regulation, snow algae such as *C. nivalis* can fix CO_2_ through photosynthesis, release oxygen, and produce organic matter (e.g., organic acid, polysaccharide) for providing a comfortable breeding environment for other native organisms. *C. nivalis* and other organisms use different metabolic modes to form a harmonious symbiosis, which promotes the recycling of limited nutrient elements, playing an important role in snow carbon cycle and energy cycle.

**FIGURE 10:**
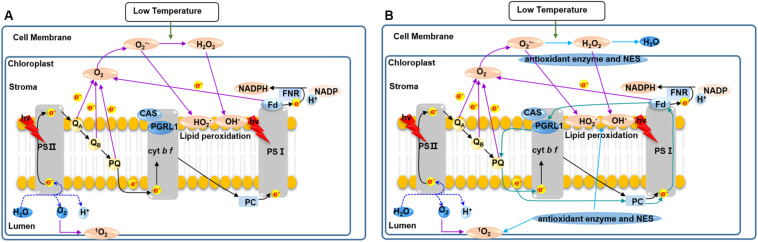
An outline of the putative response mechanisms of *C. nivalis* and *C. reinhardtii* at low temperatures. **(A)** *C. reinhardtii*. **(B)** *C. nivalis.* Black arrows, linear electron transfer. Atrovirens arrows, cyclic electron transport. Purple arrows, ROS generation. Blue arrows, ROS scavenging. NES, non-enzymatic ROS scavengers. When *C. nivalis* is exposed to low temperatures, the cyclic electron flow helps to eliminate excess light energy, while the activities of the antioxidant enzymes reduce oxidative damage.

In summary, as shown in Figure 10A photosynthesis of *C. reinhardtii* was seriously damaged by low temperature, indicated by the significantly decreased photosynthetic pigments (Chl a, Chl b, and Car) content and photosynthetic activity, and led to photo-oxidative damage to the membrane system, proteins, lipids and DNA inside the cell. As a result, *C. reinhardtii* cells failed to survive under low temperatures. As a representative snow alga, *C. nivalis* maintains a robust level of photosynthetic activity under low temperatures, promoting its survival and cellular growth. The possible adaptive mechanisms responsible for this are displayed in Figure 10B. Under low temperatures, two main processes promote photosystem fitness and maintained cellular growth in *C. nivalis*. The light-harvesting ability of PSI and PSII are reduced, which decreases the damage to the photosystem caused by the accumulation of excess light energy. Furthermore, the excess light energy is consumed by CET around PSI, which produces ATP for cellular growth and other physiological processes. Simultaneously, the excess ROS originating from the transport of electrons (from the photosynthesis pathway or other metabolic pathways) to O_2_ were eliminated by the CET-mediated regulation of the ATP/NADPH balance, carotenoids, and antioxidant enzymes, all of which prevent the oxidative damage of intracellular membrane systems, proteins, lipids, and DNA. These adaptive mechanisms related to photosynthetic regulation not only promote the survival and even blooming of *C. nivalis* under polar environment, but also provide organic carbon sources for the surrounding polar organisms and contribute to the maintenance of polar ecosystems.

## Data Availability Statement

All datasets generated for this study are included in the article/Supplementary Material.

## Author Contributions

YZ and QW designed the study. YZ, CX, HC, CH, and QW collected, analyzed, and interpreted the data. YZ, HC, and QW wrote the manuscript.

## Conflict of Interest

The authors declare that the research was conducted in the absence of any commercial or financial relationships that could be construed as a potential conflict of interest.

## Footnotes

1 http://www.chlamycollection.org/

2 https://utex.org/
