# Corrigendum: Low-Temperature Adaptation of the Snow Alga *Chlamydomonas nivalis* Is Associated With the Photosynthetic System Regulatory Process

**DOI:** 10.3389/fmicb.2021.698706

**Published:** 2021-05-07

**Authors:** Yanli Zheng, Chunling Xue, Hui Chen, Chenliu He, Qiang Wang

**Affiliations:** ^1^Key Laboratory of Algal Biology, Institute of Hydrobiology, Chinese Academy of Sciences, Wuhan, China; ^2^University of Chinese Academy of Sciences, Beijing, China; ^3^State Key Laboratory of Crop Stress Adaptation and Improvement, School of Life Sciences, Henan University, Kaifeng, China; ^4^Innovation Academy for Seed Design, Chinese Academy of Sciences, Wuhan, China

**Keywords:** antioxidant enzymes, *Chlamydomonas nivalis*, cyclic electron transfer, low temperature, photosynthesis

In the original article, there was an error in affiliation 2 as published. Instead of University of the Chinese Academy of Sciences, Beijing, China, it should be University of Chinese Academy of Sciences, Beijing, China.

The authors apologize for this error and state that this does not change the scientific conclusions of the article in any way. The original article has been updated.

