# Supplementary material for: Low-Temperature Adaptation of the Snow Alga Chlamydomonas nivalis Is Associated With the Photosynthetic System Regulatory Process
Source: Front Microbiol. 2020 Jun 10;11:1233. doi: 10.3389/fmicb.2020.01233 (PMC7297934; doi:10.3389/fmicb.2020.01233)
Supplement: Supplementary file 1 [file Data_Sheet_1.docx]

**Table S1.** Specific primers used for quantitative real-time PCR analysis

| Gene | Primer sequence (5′–3′) | *C. reinhardtii* database matches [Pytozome locus name or GenBank Accession Number*] |
| --- | --- | --- |
| *CAT1* | \| CCGCGAGACGTATGTCAAGT \| \| --- \| \| GTCTTGGTCACGTCCAGAGG \| | Cre09.g417150 |
| *CAT2* | \| TGTTCATGACGCCCTCAGAC \| \| --- \| \| GCAAAGGTCTGCAGGAAAGC \| | Cre01.g045700 |
| Fe-*SOD* | \| CCTACGCTCTGGATGCTCTG \| \| --- \| \| AGCGACCTGCTTGTTCATGT \| | Cre10.g436050 |
| Mn-*SOD1* | \| AGGATGTCGCCACTGTCATC \| \| --- \| \| ACTTGGCCTTCATCTCGTCC \| | Cre02.g096150 |
| Mn-*SOD2* | \| GCTACAACAAGGCCACACAC \| \| --- \| \| ACGTTCTGCCAGTTGACGAT \| | Cre13.g605150 |
| Mn-*SOD3* | \| GGCTTACCACTGGCTTACGA \| \| --- \| \| GACAATCTCCGACAGCGACA \| | Cre16.g676150 |
| Mn-*SOD4* | \| CTCATGGGGGTGGTGCTCAA \| \| --- \| \| GTGTAGTCCGGCTTCTTGGG \| | Cre12.g490300 |
| Mn-*SOD5* | \| CTACCTCCAGTACCAGGCCA \| \| --- \| \| GATGTTGTAGCTGAGGTCCCC \| | GU134345.1* |
| *2-cys peroxiredoxin* | \| TGGGTGAACTGGCTGTCAAT \| \| --- \| \| CCACCGAGATCACTGCCTTC \| | Cre02.g114600 |
| *2-cysperoxiredoxin, chloroplastic* | \| GGTTCTGGGTGTGTCTGTGG \| \| --- \| \| GCCCTCCTTGTCGATGATGAA \| | Cre06.g257601 |
| *Ascorbate peroxidase* | \| CAACTGGCTCGAGTTCGACA \| \| --- \| \| CATAGGGGCGGAACTGATCG \| | Cre02.g087700 |
| *Glutathione peroxidase* | \| CTGATGCGTATTGTTGTCGTTC \| \| --- \| \| AGCTACCCGGTCCTTAGAAGAG \| | Cre10.g440850 |
| *Glutathione peroxidase5* | \| GCTAGCAAGTGCGGCTTTAC \| \| --- \| \| AAATTGCGCTGGCAGAACTC \| | Cre10.g458450 |
| *Glutathione peroxidase3* | \| ACGACCTCGGAGTTCTACCA \| \| --- \| \| GCTTGCCGTACTTGTCTTGC \| | Cre03.g197750 |
| *Glutathione peroxidase1* | TGACCATCGTCGCTTTCC  CACGTCCACCTTGTCCATC | Cre02.g078300 |
| *L-ascorbate peroxidase2* | \| CAAGACGAAGACTGCTGTGG \| \| --- \| \| CGTACTGGATGGAGGCGTTC \| | Cre06.g285150 |
| *L-ascorbate peroxidase,heme-containing* | \| GTCGAAGGATGAGGCCAAGAG \| \| --- \| \| GCTGGTGAACAGTGAGTAGCC \| | g10003 |
| *Peroxiredoxin3* | \| CTGTTCGGAGGCAAGAAGGT \| \| --- \| \| ATAGGCAGGCAATGGTGTCC \| | Cre10.g449550 |
| *Peroxiredoxin5* | \| ATGCTCGCCAACATCTTCCA \| \| --- \| \| TTTACAGAGCTCGTCCGTGG \| | Cre01.g014350 |
| *Peroxiredoxin4* | \| GGACGTCTTCAAGGGCAAGA \| \| --- \| \| CCACAGTCACGCACAATACC \| | Cre02.g080900 |
| *Thioredoxin dependent peroxidase* | \| CACCGACGAGAACTCCATCC \| \| --- \| \| TTGAAGGCCATGACGCACTT \| | Cre10.g422300 |
| *18S rRNA* | \| GCCTAGTAAGCGCGAGTCAT \| \| --- \| \| AGCCAAGCTCAATCCGAACA \| | M32703* |
| *CBLP* | GCTGTGGGACCTGGCTGA  CCTTCTTGCTGGTGATGTTG | g6364 |
| *CAS* | TCAACTCGGGCACCAAGG  CGCCCACCACCACATACAC | AB127959.1 |


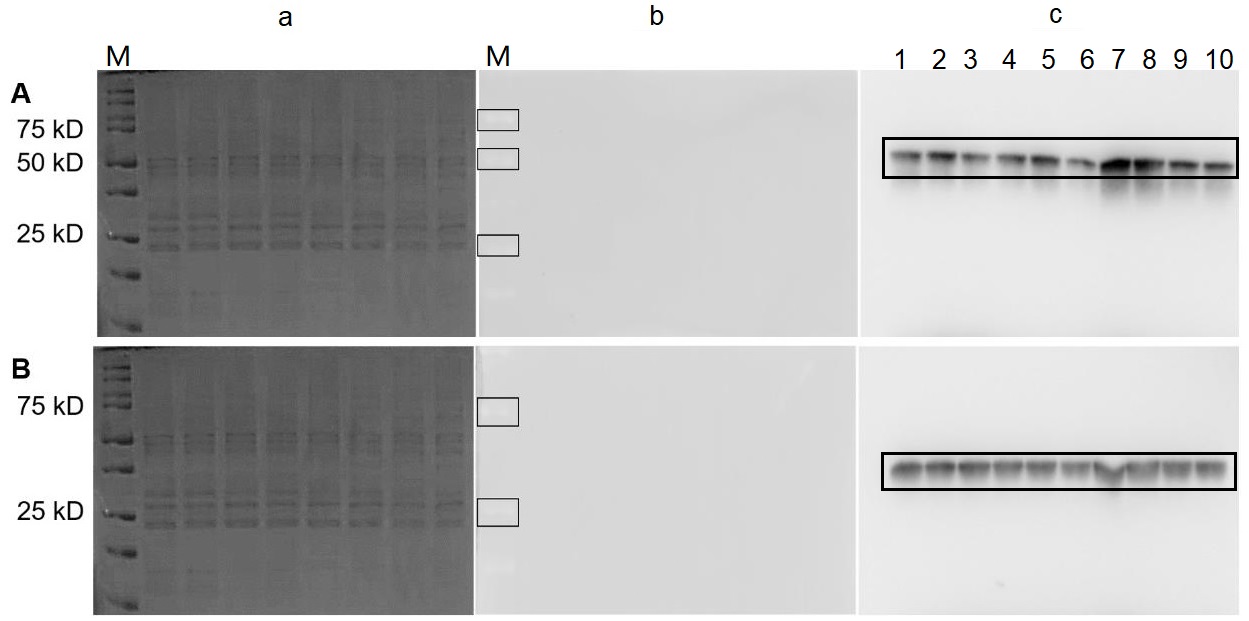


**Figure S1.** The verification of CP43 (A) and D1 (B) specific antibodies for immunoblot assays in *Chlamydomonas*. a, the representative image of SDS-PAGE analysis; b, the protein labeling (box) before chemiluminescence; c, the protein blot (bold box) of CP43 and D1 with chemiluminescence. M, protein marker. 1-5, samples collected at 12 h, 24 h, 36 h, 48 h and 60 h of regular culture; 6-10, samples collected at 12 h, 24 h, 36 h, 48 h and 60 h of N- treatment.

Antibody preparation: Preparation of both CP43 and D1 antibodies were produced in rabbits. For preparation of CP43 antibody, the nucleotide sequence encoding polypeptide sequence at 303-472aa (PTGMEASQSQAFTFLVRDQRLGANIASAQGPTGLGKYLMRSPSGEIIFGGETMRFWDFRGPWLEPLRGPNGLDLDKLRNDIQPWQVRRAAEYMTHAPLGSLNSVGGVITDVNSFNYVSPRAWLATSHFVLGFFFLVGHLWHAGRARAAAAGFEKGIDRETEPTLFMPDLD) of CP43 protein was selected and cloned into the expression vector pGEX-4T-1 for recombinant protein expression and polyclonal antibodies preparation in rabbits. By using protein Blast, only 13.8% homology between 303-472aa of CP43 protein and full amino acid sequence (LFGPTRYQWDQSYFKTEINRRVQTAMDDGATRQEAYESIPEKLAFYDYVGNSPAKGGLFRVGPMVNGDGLATSWVGHIVFTDREGRELEVRRLPNFFENFPVVLQDEQGIVRADIPYRRAEAKYSFEQQGVTAEVFGGALDGQRFTDPADVKRLARKAQLGEGFDFDRETYVP) of CP47 protein avoided the possibility of CP47 being detected by CP43 antibody. For preparation of D1 antibody, an artificially synthesized polypeptide sequence (VESQNQGYKFGQEEE) of D1protein was coupled to KLH for polyclonal antibodies preparation in rabbits.

Verification of CP43 and D1 specific antibodies: *Chlamydomonas reinhardtii* was grown in TAP medium at temperatures of 22°C with a light intensity of 100 μmol m^–2^ s^–1^ and continuously bubbled with fltered air, and the initial OD_700_ is 0.05. When cells reached the midlogarithmic growth phase (OD_700_ approximately 1.0), cells were harvested by centrifugation at 3000 *g* for 3 min at room temperature and then washed and resuspended in TAP medium and N- medium (the commonly used stress condition in our lab for verification), respectively. The N- medium used for *C. reinhardtii* was TAP without NH_4_Cl. Then, *Chlamydomonas* cells were cultured in TAP medium and N- medium with the same condition above. Samples were collected at 12 h, 24 h, 36 h, 48 h and 60 h after culture, respectively. Protein from each sample was extracted using 40 mM Tris-HCl (pH 8.0) and then adjusted to 2 mg ml^-1^ of protein concentration, quantified using a BCA Kit. 10 μl of protein extract was then used for immunoblot analysis according to the method in **Material and methods.**
